# Supplementary material for: The Synergistic Effect of Baloxavir and Neuraminidase Inhibitors against Influenza Viruses In Vitro
Source: Viruses. 2024 Sep 14;16(9):1467. doi: 10.3390/v16091467 (PMC11437495; doi:10.3390/v16091467)
Supplement: Supplementary file 1 [file viruses-16-01467-s001.zip › viruses-3177855-supplementary.pdf]

Table S1. The EC_{50} of NAIs and baloxavir against influenza resistant-strains used in this study and the relevant fold-resistance information.

Strain	Oseltamivir acid	Zanamivir	Laninamivir	Peramivir	Baloxavir
	EC_{50} (μM)	EC_{50} (μM)	EC_{50} (μM)	EC_{50} (μM)	EC_{50} (μM)
PR/8-I38T	1.96±0.30 （0.79）	2.01±0.58 （0.66）	0.13±0.07 （1.63）	0.33±0.14 （1.94）	0.670±0.09 （33.50）
PR/8-R292K	＞100 （40.16）	26.65 （8.71）	0.46±0.11 （5.75）	＞100 （588.24）	0.01±0.00 （0.50）
WSN-I38T	0.55±0.32 （4.23）	0.19±0.02 （0.70）	0.18±0.02 （0.75）	0.10±0.040 （10.00）	0.11±0.03 （78.57）

Numbers in parentheses indicate fold-resistance compared to wild-type virus.
